# Triple Synchronous Colo-Rectal Carcinoma Causing Intestinal Obstruction

**Published:** 1983-10

**Authors:** Arthur Allan

**Affiliations:** Department of Surgery, Bristol Royal Infirmary


### Triple Synchronous Colorectal Carcinoma causing Intestinal Obstruction

**Arthur Allan, M.D., F.R.C.S.**
Department of Surgery, Bristol Royal Infirmary

### INTRODUCTION

This paper reports two cases of triple synchronous colorectal cancer. They were seen within a 3 month period by one surgical team at the Bristol Royal Infirmary. Multiple colorectal tumours constitute about 6% of all such tumours, and their main characteristics are reviewed.

### CASE REPORT

*Case 1.* H.H., a 70-yr-old male, presented as an emergency with a 36 hr history of intermittent epigastric pain and absolute constipation. On examination his abdomen was distended, tender in the epigastrium, and with high-pitched bowel sounds. Plain X-rays of the abdomen indicated colonic obstruction. Laparotomy revealed three synchronous colonic tumours. One was obstructing the mid-transverse colon, and two separate tumours 6 cm apart were found at the spenic flexure. A colonic resection was performed with ileo-sigmoid anastomosis. Histologically all the tumours were moderately-differentiated adenocarcinomas. The proximal lesion at the splenic flexure was Dukes' stage A, the others were Dukes' stage B. In addition three benign tubular adenomas were found in the right colon.

*Case 2.* E.M., a 66-yr-old woman, presented as an emergency with a 4 week history of central abdominal pain and 48 hr absolute constipation. On examination her abdomen was distended, and a mass was palpable in the lower central abdomen. Plain abdominal X-rays revealed colonic obstruction and there was at operation a tumour obstructing the upper rectum. In addition there was a second tumour in the lower sigmoid colon and a third tumour in the transverse colon. A colonic resection and ileo-rectal anastomosis was carried out. Histology revealed three moderately differentiated adenocarcinomas. Both recto-sigmoid lesions were Dukes' stage C, but the transverse colonic lesion was a Dukes' stage B. A benign tubular adenoma was also found in the rectum.

### DISCUSSION

### PREDISPOSING LESIONS

The concept of colonic adenomas developing into carcinomas is well established.[1] In a review of over 4000 resection specimens for colonic cancer at St Mark's Hospital, 25% of specimens with a single carcinoma also contained an adenomatous polyp. When specimens containing multiple colonic tumours were studied, 75% were found to contain an adenomatous polyp.[2] Moreover the likelihood of developing a metachronous tumour was twice as high in those patients who had adenomas associated with their first colonic carcinoma compared with those with no such adenoma.[3] The risk of developing a colonic tumour rises proportionally with the number of adenomas in the colon,[4] and in patients with familial polyposis coli malignant change is the rule. There is a strong association therefore between adenomas and both synchronous and metachronous colonic cancers.

The frequency of colonic carcinoma amongst patients with chronic ulcerative colitis is estimated as thirty times that expected in the general population.[5] In this situation tumours are characteristically of high grade malignancy and multifocal in origin.

### SYNCHRONOUS CANCER

The incidence of synchronous colonic lesions is in the region of 3%. Table 1 summarises the findings of five large reviews of synchronous colonic tumours. The incidence of three synchronous tumours is much less, 0.25% in Moertel's series[6] and 0.2% in Heald's series.[2] There is a tendency to spatial clustering. In one study, 28% of synchronous tumours were found within the same anatomical segment of bowel and 68% within the same or adjacent segments.[6] Even so, detection before surgery is uncommon. Table 2 shows the method of detection in 175 cases of synchronous tumour reported by Heald and Bussey.[2] The pick-up rate on barium enema would undoubtedly be higher today, if the study were repeated, because many of Heald's cases were investigated before the introduction of air contrast barium studies. The prognosis of synchronous

**Table 1**

**Incidence of Synchronous Colonic Tumours**

| | *No. of cases* | *No. of multiple tumours* | *Occurrence (%)* |
|---|---|---|---|
| Moertel 1958[6] | 6012 | 166 | 2.8 |
| Heald 1975[2] | 4884 | 175 | 3.5 |
| Schottenfeld 1969[7] | 4771 | 171 | 3.6 |
| Enker 1978[8] | 3842 | 68 | 1.7 |
| Diamante 1966[9] | 2508 | 80 | 3.1 |
| | | Mean | 2.94 |

**Table 2**

**Method of Detection of 175 Cases of Synchronous Colonic Tumour (Heald 1975[2])**

| | % |
|---|---|
| Clinical detection | 2.3 |
| Sigmoidoscopy | 9.1 |
| Barium enema | 2.9 |
| Palpable at operation | 27.4 |
| Found in resection specimen | 38.0 |
| Missed to become 'Metachronous' | 10.0 |

**Table 3**

**Crude 5 Year Survival by Stage (Heald 1975[2])**

| *Duke's stage* | *No. of patients* | *Crude 5 year survival % Multiple* | *Single* |
|---|---|---|---|
| A+A | 17 | 88 | 83 |
| B+A | 43 | 60 | |
| B+B | 12 | 66 | 63 |
| C+A | 39 | 43 | 28 |
| C+B | 16 | 35 | |
| C+C | 4 | 25 | |

**Table 4**

**Relationship between Length of Follow up and Development of Metachronous Tumours (Bussey 1967[3])**

| *Group* | *Period of resection* | *No. of patients* | *No. of second cancer* | *%* |
|---|---|---|---|---|
| 1 | 1928–32 | 217 | 9 | 3.65 |
| 2 | 1933–37 | 321 | 9 | 2.80 |
| 3 | 1938–42 | 381 | 7 | 1.83 |
| 4 | 1943–47 | 584 | 5 | 0.85 |
| 5 | 1948–52 | 1025 | 18 | 1.75 |
| 6 | 1953–57 | 853 | 0 | 0.70 |

colonic tumours is surprisingly good, and is determined by the Dukes' stage of the least favourable tumour. Table 3 shows the crude five year survival rate of 131 patients studied at St Mark's Hospital.[2] The survival rate for similar single tumours over the same years at the hospital are shown for comparison. Cases of triple synchronous carcinoma show the same favourable trend.[2] When synchronous tumours exist, the risk of developing a metachronous tumour within 30 years reaches 8%,[2] and it has been suggested that for patients below 50 years of age, subtotal colectomy and ileo-sigmoid anastomosis is the procedure of choice.

### METACHRONOUS CANCER

The chances of an individual with a colorectal carcinoma developing a metachronous tumour increase with time. Table 4 gives the incidence of metachronous tumour development in 3381 patients followed up for 30 years at St Mark's Hospital.[3] Since nearly all the patient in the 1928–32 group are dead, the true incidence is probably in the region of 3.6%. It has been shown that the stage and grade of the first tumour is often favourable, probably because there are more long term survivors from operations for less advanced tumours.[3] There is no correlation between the histological grade and stage of the first and second tumours.[10] The prognosis of patients with a metachronous tumour is similar to the prognosis of a patient with a single tumour of the same grade and stage.

### PROPHYLAXIS

What can be done to minimise the chance of missing multiple colonic tumours? All cases of colorectal tumours for elective surgical resection should have a good quality air contrast barium enema examination before operation. Patients found to have a synchronous polyp should in addition be colonoscoped, the polyp removed for histological examination, and any other small synchronous tumour or polyps excluded. At operation the bowel should be carefully palpated for a second tumour, and the resection specimen should be opened in the theatre suite. Synchronous tumours may be easily overlooked during emergency colonic resections for obstructing lesions. In these cases the distended bowel is more difficult to palpate, and thorough preoperative investigation is impractical. A preoperative sigmoidoscopy is useful, and after operation the colon should be investigated either by barium enema or by colonscopy, particularly in those patients with a colostomy.

Patients who undergo a resection for a colonic tumour are generally followed up annually in the clinic with sigmoidoscopy and faecal occult blood testing, although the value of this follow up has been questioned.[11,12] Further investigations are only performed on suspicion of a second lesion. These patients do form a group at increased risk of a second tumour and should ideally have a barium enema or colonoscopy annually. This strategy is probably impractical and is best reserved for patients with multiple colonic adenomas, a tumour with synchronous poylps, or multiple tumours. Patients with a single adenoma should be colonoscoped annually until no further polyps are found. Following this colonscopy every five years is probably all that is required.[13]

Finally it should be remembered that the prognosis of multiple colonic tumours is as good as for comparable single tumours and an unduly conservative policy in treating them is not indicated. Younger patients with synchronous lesions are probably best treated by an extended colonic resection and ileosigmoid anastomosis.

### ACKNOWLEDGEMENT

I am grateful to Professor R. C. N. Williamson for permission to report his cases.

### REFERENCES

1. MORSON, B. C. (1974) The polyp-cancer sequence in the large bowel. *Proc.R.Soc.Med.* **67**, 451.
2. HEALD, R. J. and BUSSEY H. J. R. (1975) Clinical experiences at St Mark's Hospital with multiple synchronous cancers of the colon and rectum. *Dis.Col.Rect.* **18**, 6.
3. BUSSEY, H. J. R., WALLACE, M. M. and MORSON, B. C. (1967) Metachronous carcinoma of the large intestine and intestinal polyps. *Proc.R.Soc.Med.* **60**, 208.
4. BUSSEY, H. J. R. (1978) 'Multiple adenomas and carcinomas' in the pathogenesis of colorectal cancer. Major Problems in Pathology 10. London, Saunders, p. 72.
5. BARGEN, J. A., SAUER, W. G., SLOAN, W. P. and GAGE, R. P. (1953) The development of cancer in chronic ulcerative colitis. *Gastroenterology* **26**, 32.
6. MOERTEL, C. G., BARGEN, J. A., DOCHERTY, M. B. (1978) Multiple carcinomas of the large intestine. *Gastroenterology* **34**, 85.
7. SCHOTTENFELD, D., BERG, J. W. and VITSKY, B. (1969) Incidence of multiple primary cancers II. Index cancers arising in the stomach and lower digestive system. *J.Nat.Cancer Institute* **43**, 77.
8. ENKER, W. E. and DRAGACEVIC, S. (1978) Multiple carcinomas of the large bowel. *Ann.Surg.* **187**, 8.
9. DIAMANTE, M. and BACON H. E. (1966) Primary multiple malignancy of the colon and rectum. *Dis.Colon.Rect.* **9**, 441.
10. AGREZ, M. V., READY, R., ILSTRUP, D. and BEART, R. W. (1982) Metachronous colorectal malignancies. *Dis.Colon.Rect.* **25**, 569.
11. COCHRANE, J. P. S., WILLIAMS, J. T., FABER, R. G. and SLACK, W. W. (1980) Value of outpatient follow up after curative surgery for carcinoma of the large bowel. *Br.Med.J.* **280**, 593.
12. ROSS, A. P. J. (1983) A time saving but effective approach to the follow up of patients after curative surgery for carcinoma of the large bowel. *Annals.R.Coll. Surg.Engl.* **65**, 8.
13. MINOPOULOS, G. J., McINTYRE, R. L. E., LEE, E. C. G. and KETTLEWELL M. G. W. (1983) Colonoscopic polypectomy in a regional teaching hospital. *Br.J.Surg.* **70**, 51.

